# Please Take Notice

**Published:** 1873-03

**Authors:** 


*Please Take Notice.*

We receive letters quite frequently well and correctly written in every respect, save only that the names of the writers cannot be deciphered. Often the post-office, State and county are not mentioned. Correspondents will oblige us by writing their names *plainly*, and also by giving the *State, county* and *post-office.* Please remember this.

